# Inhibiting Caveolin-1-Related Akt/mTOR Signaling Pathway Protects Against N-methyl-D-Aspartate Receptor Activation-Mediated Dysfunction of Blood–Brain Barrier in vitro

**DOI:** 10.1007/s12035-023-03833-7

**Published:** 2023-12-08

**Authors:** Fang Huang, Fengping Mao, Weidong Nong, Zhuowei Gong, Dayuan Lao, Wen Huang

**Affiliations:** Department of Neurology, the First Affiliated Hospital of Guangxi Medical University, #6 Shuangyong Road, Nanning, 530021 Guangxi China

**Keywords:** Brain microvascular endothelial cells, N-methyl-D-aspartate receptor, Caveolin-1, Matrix metalloproteinase 9, Occludin

## Abstract

**Background:**

The aim of this study was to further explore the role of caveolin-1 (Cav-1) related Akt/mTOR signaling pathway in blood brain barrier (BBB) dysfunction caused by NMDAR activation.

**Methods:**

The cell localization of NMDAR GluN1 subunit and Cav-1 was observed on human brain microvascular HBEC-5i cells after immunofluorescence double staining. The transendothelial resistance (TEER) of BBB in vitro was measured by Millicell-ERS cell resistance meter. Sodium fluorescein (SF) was used to measure the permeability of BBB in vitro. A stable Cav-1-silenced HBEC-5i cell line was established by infecting the cells with a lentivirus encoding Cav-1 shRNA. The changes of the protein and mRNA of MMP9 and Occludin induced by NMDA were detected by Western blot (WB) and real-time quantitative reverse transcription polymerase chain reaction (qRT-PCR), respectively. The phosphorylated proteins of Cav-1, Akt, and mTOR were detected by WB.

**Results:**

NMDAR GluN1 was expressed in the cytoplasm and part of the cell membrane of the HBEC-5i cell line. NMDAR activation decreased TEER and increased the SF of BBB in vitro. HBEC-5i cells incubated with NMDA enhanced the phosphorylation of Cav-1, Akt, and mTOR, also promoting the expression of MMP9 along with the degradation of Occludin. These effects could be reversed by pretreatment with NMDAR antagonist (MK801) or Cav-1 antagonist (Daidzein), or Akt antagonist (LY294002), respectively. Further silencing Cav-1 with LV-Cav-1-RNAi also played a similar protective effect.

**Conclusion:**

Caveolin-1 (Cav-1) related Akt/mTOR signaling probably contributes to BBB dysfunction by activating NMDAR on human brain microvascular cells.

**Supplementary Information:**

The online version contains supplementary material available at 10.1007/s12035-023-03833-7.

## Introduction

NMDAR (N-methyl-D-aspartate receptor) is an ionic glutamate receptor expressed in the postsynaptic membrane of excitatory synapses of neurons in the central nervous system (CNS). It is mainly distributed in the prefrontal cortex, hippocampus, amygdala, hypothalamus, and spinal cord. NMDAR is assembled from two intrinsic GluN1 A-B subunits and two optional regulatory GluN2 A-D or/and GluN3 A-B. GluN1 is one of the subunits of the heterotetrameric cationic complex, which is necessary for neurogenesis and cell survival [1]. Thus, antibodies against GluN1 potentially affect all NMDARs in the CNS. The NMDAR plays an important role in learning and memory by participating in various synaptic transmissions and plasticity regulation [2]. In addition, multiple studies have shown that glutamate and its analogue NMDA can damage blood–brain barrier (BBB) function through excitatory toxic reaction produced by over activating NMDARs on cerebral microvascular endothelial cells [3, 4].

The BBB is a physical barrier between blood and the central nervous system, which plays a key role in maintaining the homeostasis of brain environment [5]. BBB destruction is highly related to a variety of CNS diseases, such as ischemic stroke [6], epilepsy [7], Alzheimer’s disease [8], and anti-NMDAR antibody encephalitis [9]. The anatomical basis of the BBB is related to the brain microvascular endothelium, which forms “neurovascular units” together with astrocytes, pericytes, neurons, and extracellular stroma [10]. Compared with endothelial cells of other peripheral organs, there are unique and more widely distributed tight junctions (TJs) between cerebrovascular endothelial cells [11]. This transmembrane protein complex is composed of more than 40 proteins such as Occludin, claudin-5 and ZO-1. The biological barrier forms a high impedance physical barrier, which makes it difficult for most blood derived substances and peripheral immune cells to enter through the paracellular pathway of the BBB [12]. Occludin is not only a key player in transepithelial electrical barrier function, but also a significant structural element in the construction of aqueous pores within TJ strands [13]. Matrix metalloproteinase9 (MMP9) is mainly located in neurons, vascular endothelial cells, and glial cells [14]. It is secreted to the extracellular environment in the form of zymogen and can be activated under inflammation. Activated MMP9 can directly degrade the extracellular matrix components of the basement membrane and TJs. In addition, MMP9 affects TJ gene expression by suppressing the Hedgehog pathway in brain endothelial cells [15].

Caveolin is both a positive and negative regulator of cell signaling in and/or out of caveolae, which is an invaginated lipid raft domain whose formation is dependent on caveolin expression [16]. Caveolin-1 (Cav-1) is the main marker of caveolae in endothelial cells. It was found to play different functions in the BBB in different nervous system diseases and cell events by regulating the expression of MMP9 [17], which affects the degradation and redistribution of TJs (Occludin, claudin-5) [18]. Our previous studies have shown that NMDA exposure increased intracellular reactive oxygen species (ROS) in human brain microvascular endothelial cells (HBMECs), which promoted apoptosis and finally destroyed the BBB [4]. The Akt/mTOR pathway is one of the most classic pathways regulating biological processes such as cell proliferation, differentiation, and apoptosis. This pathway has been confirmed in some recent studies to participate in the regulation of BBB integrity by regulating autophagy and ROS production, in addition to inducing Occludin degradation [19, 20]. However, it has not been reported whether Akt/mTOR is directly involved in the changes of BBB function caused by NMDA induced NMDAR activation. Our previous studies found that Cav-1 knockout reduced NMDA-mediated BBB dysfunction by reducing ERK1/2 phosphorylation [21]. However, it is not very clear whether the signaling cascade after NMDAR activation affects the change of Akt/mTOR through Cav-1. Therefore, this study is focused on evaluating the role of Cav-1-related Akt/mTOR signaling pathway in the activation of NMDAR-mediated dysfunction of the BBB in vitro. Our results showed that NMDAR activation increased the phosphorylation of Cav-1, Akt, and mTOR. It also promoted the expression of MMP9 and the degradation of Occludin. Pretreated with MK801, Cav-1 and Akt respectively could reverse these effects to protect the BBB. Further silencing Cav-1 had similar protective effects.

## Materials and Methods

### Cell Cultures

The monoculture model with Human cerebral microvascular endothelial cells (HBEC-5i) (from ATCC-Manassas, VA, USA) was conducted as described earlier [22]. HBEC-5i cells can grow monolayer on the polycarbonate membrane between the upper and lower compartments of Transwell plate, becoming an ideal BBB model in vitro with high TEER value and low permeability. The cells were cultured on 0.1% gelatin solution-coated (ATCC) flasks in Dulbecco's Modified Eagle Medium (DMEM)/F-12 medium (ATCC). They were supplemented with 10% fetal bovine serum (FBS, Gibco/Thermo Fisher, Waltham, MA, USA), 40 μg/mL endothelial cell growth supplement (ECGS, ATCC), 100 U/mL penicillin, and 0.1 mg/mL streptomycin (Beyotime, Shanghai, China).

### Lentivirus-Mediated Cav-1 RNA Interference

We established a stable Cav-1-silenced HBEC-5i cell line by infecting the cells with a lentivirus encoding Cav-1 shRNA (GenePharma, Shanghai, China) as before [21]. The sequence of targeted-Cav-1-RNAi used was ccACCTTCACTGTGACGAAAT. The negative vector contained a nonsense shRNA (5’- TTCTCCGAACGTGTCACGT -3’) to control for any non-RNAi-mediated effects. After synthesizing polymerase chain reaction (PCR) products based on these designs, they were cloned into the central part of a GV493 lentiviral vector. T293 cells were transfected with the packaging plasmid using GV493 for 48 h. The lentiviruses were then harvested with a collection of the supernatants, followed by concentrations and viral titers measurement. HBEC-5i cells were exposed to lentivirus-containing supernatant for 12 h. Stable transfectants were selected with puromycin (1 μg/ml) for 6 days and verified by real-time PCR.

### Confocal Microscopic Analyses of GluN1 and Cav-1

HBEC-5i cells grown on glass bottom cell culture dishes were fixed in 4% paraformaldehyde for 30 min, rinsed, permeabilized with 0.3% Triton X-100, rinsed a second time, and incubated with 3% bovine serum albumin in phosphate-buffered saline for 1 h. For double staining of GluN1 (1: 100, mouse monoclonal, #32–0500 in Thermo Fisher, Waltham, USA) and Cav-1(1: 400, rabbit monoclonal, #3267 T in CST, Boston, USA), cells were immunolabeled with a mixture of monoclonal anti-GluN1 and anti-Cav-1antibodies, washed, and treated with a mixture of Anti-rabbit IgG (H + L) F(ab')2 Fragment (Alexa Fluor®594 Conjugate, 1:500, #8889S in CST, Boston, USA) and Anti-mouse IgG (H + L) F(ab')2 Fragment (Alexa Fluor® 488 Conjugate, 1:1000, #4370 T in CST, Boston, USA). Images were obtained using Leica TCS SP8 Confocal Laser microscope.

### Experimental Design and Accessments of BBB Integrity in vitro

**Fig. 1:**
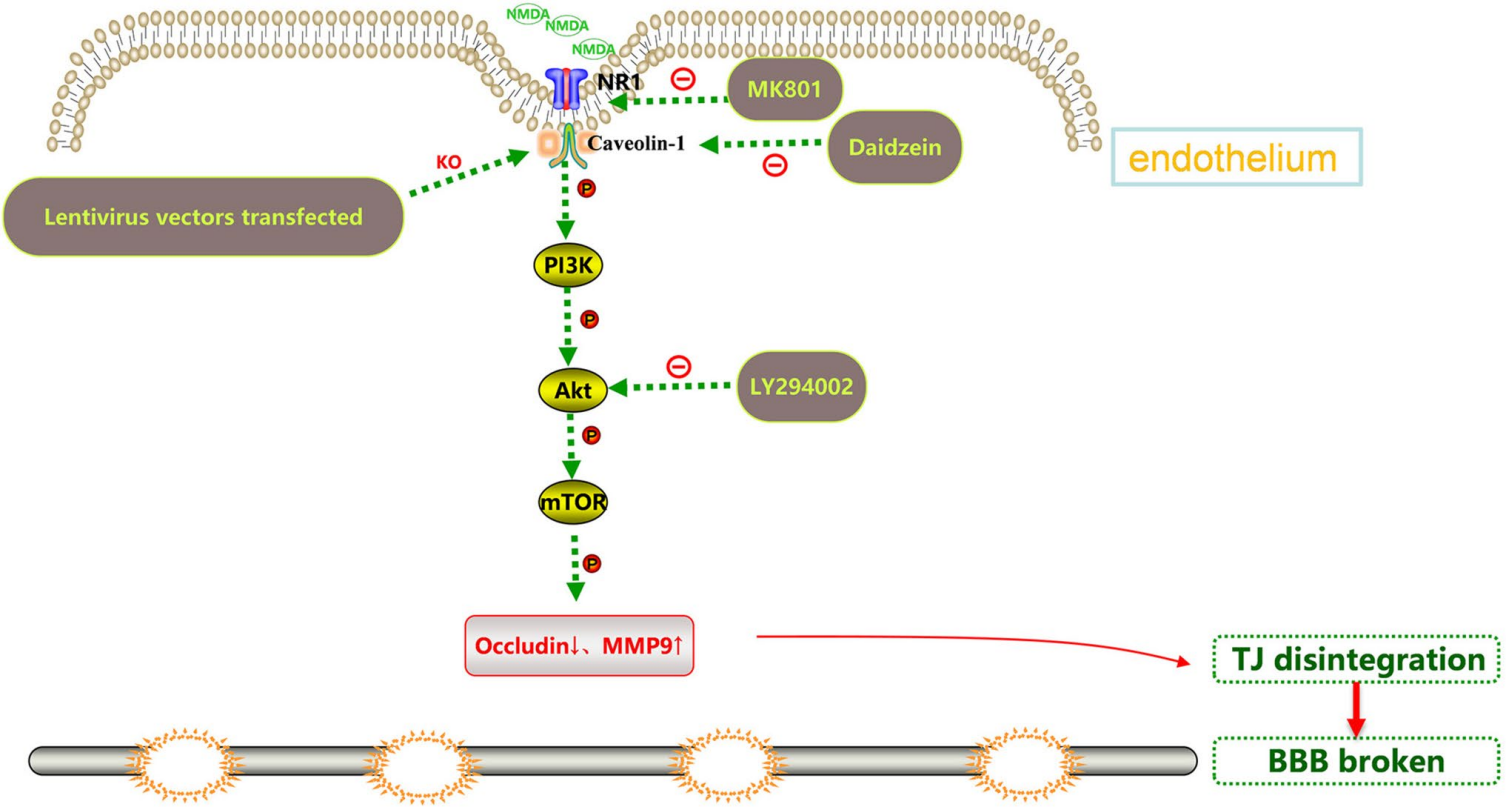
Schematic diagram of different site intervention of Cav-1 related Akt/mTOR signal pathway

The interventions were carried out according to the following groups: 1) Blank control group; 2) NMDA (2.5 mM, HY-17551, MedChem Express, USA) group: HBEC-5i were incubated with NMDA alone for 24 h; 3) MK801 (10 μM, HY-15084B, MedChem Express, USA)/Daidzein (10 μM, HY-N0019, MedChem Express, USA)/LY294002 (10 μM, HY-10108, MedChem Express, USA) group: HBEC-5i were incubated with MK801/Daidzein/LY294002 for 24 h; 4) MK801/Daidzein/LY294002 + NMDA group: HBEC-5i cells were pretreated with MK801/Daidzein/LY294002 for 2 h, then incubated with NMDA for 24 h. The selected concentration of the drugs and their exposure time were confirmed in our previous study and other research literatures [21, 23–25]. The effects of MK801, Daidzein and LY294002 on the viability of HBEC-5i were assessed by a Cell Counting Kit-8 assay (Dojindo, Kumamoto, Japan) (shown in supplementary data). To further study the role of Cav-1 in the change of the Akt/mTOR pathway after NMDAR activation, the transfected cells were randomly grouped as followed: 1) LV-Ctrl shRNA: HBEC-5i were transduced with Ctrl shRNA lentiviral vectors; 2) NMDA + LV-Ctrl shRNA: HBEC-5i were transduced with Ctrl shRNA lentiviral vectors and incubated with NMDA for 24 h; 3) LV-Cav-1 sh RNA: HBEC-5i were transduced with Cav-1 shRNA lentiviral vectors; 4) NMDA + LV-Cav-1 shRNA: HBEC-5i were transduced with Cav-1 shRNA lentiviral vectors and incubated with NMDA for 24 h. Schematic diagram of different site intervention of Cav-1 related Akt/mTOR signal pathway is shown in Fig. 1.

The integrity of the BBB model was confirmed by measuring transendothelial electrical resistance (TEER) and using sodium fluorescein (SF) permeability assay. TEER values of monolayers of HBEC-5i were seeded on transwells then cultured for 2–3 days (the TEER value reached a plateau phase, TEER > 90 Ω*cm^2^) using an TEER measurement system -EVOM (ERS-2, Millipore, Burlington, MA, USA) [26]. Each TEER value was calculated by subtracting the resistance (Ω) value of a blank transwell and multiplying by the transwell surface area. SF permeability assay was measured as previously described [27]. Treated BBB models on cell culture inserts were tested for the flux of SF (10 µg/ml) in D-hanks solution. Meanwhile, the flux of cell-free inserts was measured to calculate the endothelial permeability coefficient (Pe).

### Quantitative Real-Time Polymerase Chain Reaction (qRT-PCR)

Total RNA was extracted using the NucleoZol kit (MACHEREY–NAGEL, Germany). Then, 2 µg RNA was used for reverse transcription. The process of quantitative real-time PCR was performed in the Step One Plus Real-Time PCR System (Applied Biosystems, Foster City, CA, USA). Relative gene expression was determined by the fluorescence intensity ratio of the target gene to GAPDH. Every group was repeated three times with a similar result. Primers were as follows:

- Occludin F:5’-TCAGGGAATATCCACCTATCACTTCAG-3’.
- R:5’-CATCAGCAGCAGCCATGTACTCTTCAC)
- MMP9 F:5’-CCCTGGTCCTGGTGCTCCTG-3’
- R:5’-CTGCCTGTCGGTGAGATTGGTTC-3’
- Cav-1 F:5’-GCAGAACCAGAAGGGACACACAG-3’.
- R:5’-CCAAAGAGGGCAGACAGCAAGC-3’
- GAPDH F:5’-CAGGAGGCATTGCTGATGAT-3’
- R:5’-GAAGGCTGGGGCTATTT-3'

### Western Blot Analysis

Treated cells were prepared in Radio Immunoprecipitation Assay (RIPA) lysis buffer (Beyotime) for 30 min. After centrifugation at 12,000 rpm for 15 min, the supernatants were collected and protein concentrations were determined using BCA Protein Assay Kit (Beyotime). Samples were separated on SDS-PAGE gel, blotted onto PVDF membranes, then incubated with the respective primary antibodies at 4 °C overnight: GluN1 (1: 1000, mouse monoclonal, #32–0500 in Thermo Fisher, Waltham, USA), Occludin (1: 800, rabbit polyclonal, #27,260–1-AP in Proteintech Group, Chicago, IL, USA), MMP9 (1:800, #10,375–2-AP in Proteintech Group, Chicago, IL, USA), Cav-1 (1: 1000, rabbit monoclonal, #3267 T in CST, Boston, USA), p-Cav-1 (1: 1000, rabbit polyclonal, #3251 T in CST, Boston, USA), Akt (1: 1000, rabbit monoclonal, #4691 in CST, Boston, USA), p-Akt (1: 2000, rabbit monoclonal, #4060S in CST, Boston, USA), mTOR (1: 1000, rabbit monoclonal, #2983 in CST, Boston, USA), p-mTOR (1: 1000, rabbit monoclonal, #5536 in CST, Boston, USA), and GAPDH (1: 5000; rabbit polyclonal, 10,494–1-AP in Proteintech Group, Chicago, IL, USA). Then, samples were incubated with goat anti-rabbit fluorescent secondary antibody (1: 10,000; PA5-17,447 in Thermo Fisher Scientific) for 1 h at room temperature. The detected proteins were scanned and captured with the Odyssey Infrared Imaging System (LI-COR Biosciences). Bands were analyzed by ImageJ software (National Institutes of Health, Bethesda, MD, USA).

### Statistical Analysis

All data were analyzed using SPSS version 22.0 (SPSS, Chicago, IL, USA) or GraphPad Prism software (version 9.3). The results were displayed as mean ± standard deviation (SD) and results were obtained from at least three separately performed experiments. One way ANOVA test was performed to determine whether there were significant differences between groups. *P* < 0.05 was considered to indicate statistical significance.

## Results

### Expressions of GluN1 and Cav-1 in HBEC-5i Cells

**Fig. 2:**
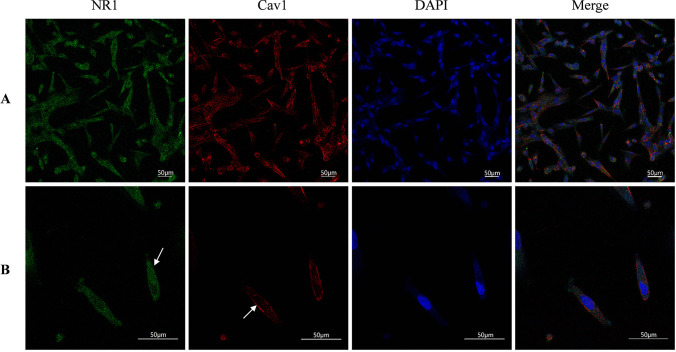
The expressions of Cav-1 and GluN1 in HBEC-5i cells. GluN1 is expressed in the cytoplasm and part of the membrane of HBEC-5i cells, and Cav-1 mainly in the membrane as detected by confocal microscope after double immunofluorescence staining. Magnification × 20 (**A**) and magnification × 60 (**B**), GluN1 subunit is green (mainly cytoplasmic), Cav-1 is red (mainly cell membrane), and the nuclei is stained in blue with DAPI

NMDARs are widely expressed in most types of neurons. Besides neurons, more and more studies have confirmed the presence of NMDAR in endothelial cells in the central nervous system. GluN1 expression was first detected in HBEC-5i in our previous study by Western blot [21]. Cav-1, as the main component of Caveolae, is generally expressed in most cells such as adipocytes and various epithelial cells, especially in endothelial cells [28]. To gain insight on the relationship between NMDAR activation and the Cav-1-associated pathway, we first employed immunofluorescence double staining to observe the co-expression and cellular distribution of GluN1 and Cav-1 in HBEC-5i cells. As shown in Fig. 2, GluN1 is expressed in the cytoplasm and membrane of HBEC-5i cells, this is consistent with the findings observed by Kim et al. in primary brain vascular endothelial cells [29]. As shown in a previous study, Cav-1 is mainly expressed in the membrane. Magnification × 20 (Fig. 2A) and × 60 (2B).

### Effects of Cav-1-Related Akt/mTOR Pathways in NMDA-Induced changes in MMP9 and Occludin

**Fig. 3:**
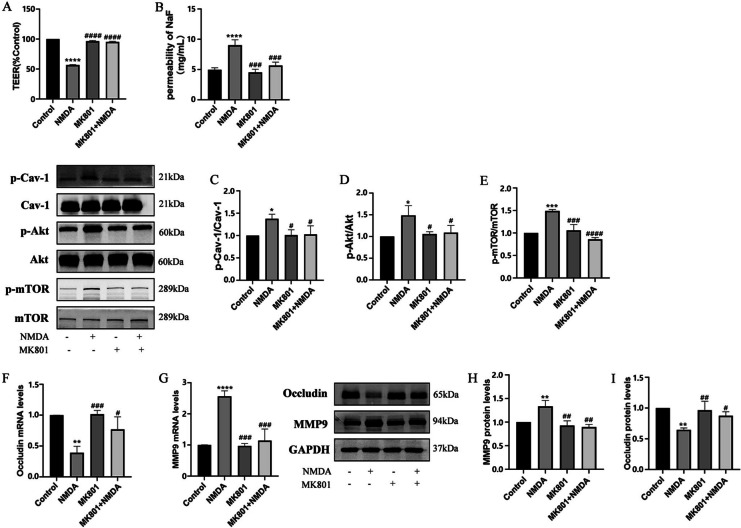
NMDA-induced changes in TEER and SF flux of BBB model in vitro and expressions of MMP9 and Occludin via the phosphorylation of Cav-1 related Akt/mTOR pathway. The transendothelial electrical resistance (TEER) value decreased after exposure to 2.5 mM NMDA (NMDAR agonist) for 24 h and was restored by pretreatment with 10 μM NMDAR antagonist MK801 for 2 h (**A**). NMDA-induced increase SF flux was also reversed by pretreatment with MK801 (**B**). Expression of phosphorylated Cav-1 (**C**), Akt (**D**) and mTOR (**E**) were immunodetected, and total protein were used as the internal standard. The mRNA levels of MMP9 (**F**) and Occludin (**G**) were detected by RT-PCR. Levels of protein of MMP9 (**H**) and Occludin (**I**) were measured by Western blot. Each value represents the mean ± SD for (*n* = 4). *p < 0.05, **p < 0.01, ***p < 0.001, ****p < 0.0001 versus control group; #*p* < 0.05, ##*p* < 0.01, ###*p* < 0.001, ####*p* < 0.001 versus NMDA group

The excitotoxic effects induced by excessive glutamate are primarily mediated by activation of NMDAR [30]. To verify the effects of NMDAR activation on the integrity of the BBB, TEER and the flux of SF were measured after exposure to 2.5 mM NMDA (NMDAR agonist) for 24 h as described previously [21]. As showed in Fig. 3A and 3B, treatment with NMDA significantly reduced TEER and increased SF in comparison with the control group. However, pretreatment with NMDAR antagonist-MK801 (10 μM) for 2 h and co-exposure to NMDA for 24 h reduced NMDA-induced BBB damage.

It has been suggested that phosphorylation of Cav-1 may contribute early BBB breakdown [31]. NMDA induced neurotoxicity is directly related to the activation of the Akt/mTOR signaling pathway [32]. The relationship between the disruption of the BBB caused by activated endothelial NMDARs and Cav-1-related Akt/mTOR pathway remains unclear. To confirm this, phosphorylation of Cav-1, Akt, and mTOR were analyzed after HBEC-5i was incubated with 2.5 mM NMDA for 24 h. Compared to the control group, the phosphorylation levels of Cav-1, Akt, and mTOR in the group with NMDA exposure increased significantly, but the total amount of protein did not change. Pretreatment with MK801 alleviated the phosphorylation of Cav-1, Akt, and mTOR triggered by NMDA (Fig. 3C, D and E).

Endothelial NMDAR activation could disrupt BBB function through various mechanisms, including altering the expression or redistribution of TJs and modulating the release of MMP9 [23]. To evaluate the changes of TJs in HBEC-5i cells caused by NMDAR activation, the amount of MMP9 and Occludin was recorded. Compared to the control group, NMDA exposure increased both mRNA and protein levels of MMP9, while causing a reduction in the mRNA and protein levels of Occludin. Pretreatment with MK801 reduced NMDA-induced increase of MMP9 and degradation of Occludin (Fig. 3F, G, H and I).

Thus, NMDA-induced excitotoxicity was accompanied with of down-regulation Occludin and up-regulation of MMP9. This effect might require activation of Cav-1-related Akt/mTOR pathways.

### Inhibition or Depletion of Cav-1 Alleviates NMDA-induced BBB Destruction through the Akt/mTOR Pathway

**Fig. 4:**
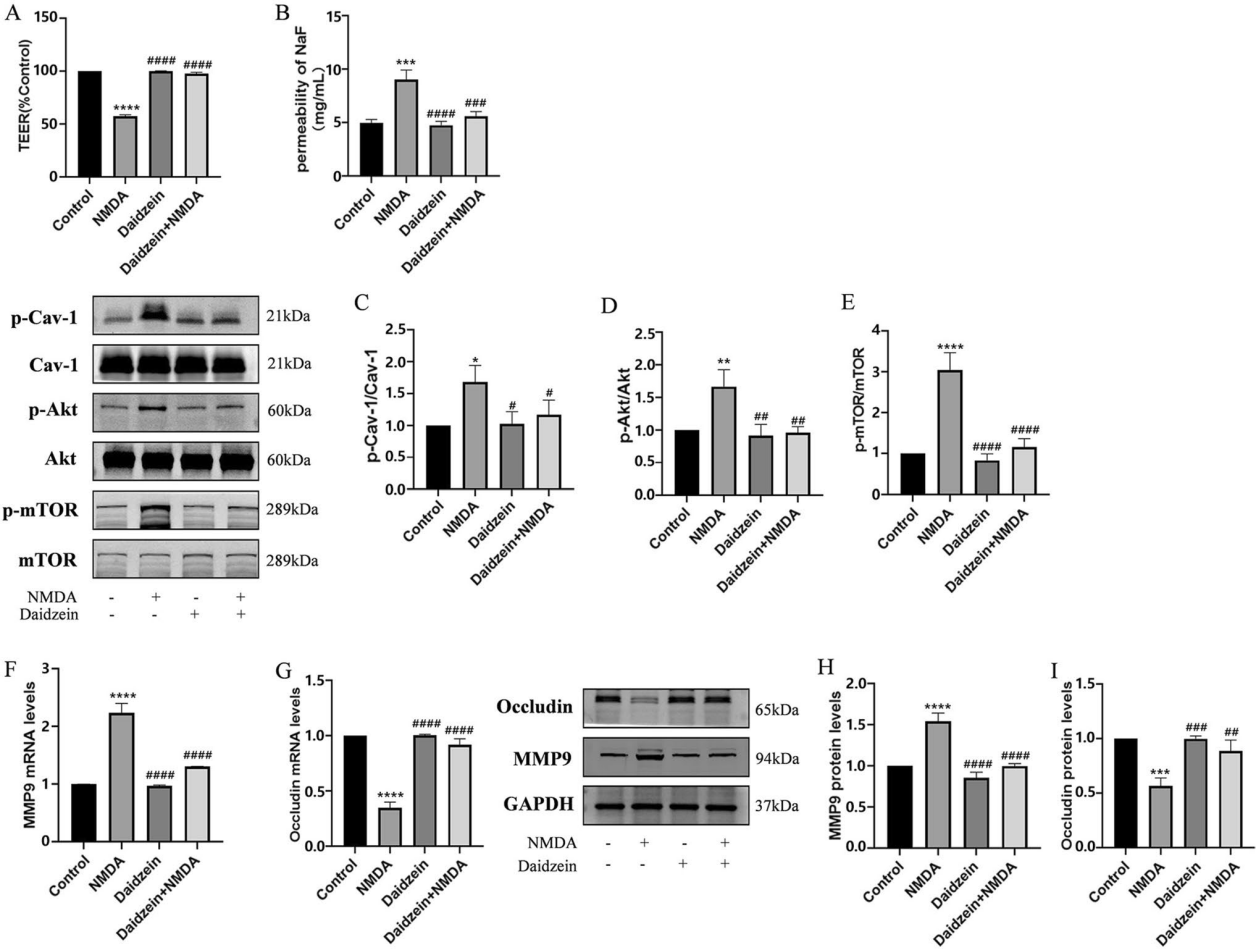
Inhibition of Cav-1 reversed NMDA-induced changes in TEER and SF flux of the BBB model in vitro and expressions of MMP9 and Occludin through Cav-1 related Akt/mTOR signal pathway*.* Pretreatment with Cav-1 inhibitor Daidzein (10 μM) for 2 h could restore decreased TEER after exposed to NMDA (**A**). NMDA-induced increase Sodium fluorescein flux was also reversed by Daidzein (**B**). Protein expressions of p-Cav-1/Cav-1 (**C**), p-Akt/Akt (**D**) and p-mTOR/mTOR (**E**) were detected by Western blot assay. The mRNA and protein levels of MMP9 (F and H) and Occludin (G and I) were detected by RT-PCR and by Western blot, respectively. Each value represents the mean ± SD (*n* = 4). *p < 0.05, **p < 0.01, ***p < 0.001, ****p < 0.0001 versus control group; #*p* < 0.05, ##*p* < 0.01, ###*p* < 0.001, ####*p* < 0.001 versus NMDA group

Downregulation of Cav-1 expression likely helped to maintain BBB integrity and normalized the expression levels of TJ proteins [33]. To investigate a possible involvement of Cav-1 in NMDA-induced BBB dysfunction, HBEC-5i cells were pretreated with Cav-1 inhibitor (Daidzein, 10 μM) for 2 h and then co-exposed to NMDA for 24 h. NMDA-induced disruption of BBB integrity was blocked by Daidzein (Fig. 4A and B). Daidzein directly reduced the phosphorylation of Cav-1 and NMDA-induced phosphorylation of Akt and mTOR (Fig. 4C, D and E), leading to reduction in MMP9 expression and increase in Occludin expression (Fig. 4F, G, H and I).

**Fig. 5:**
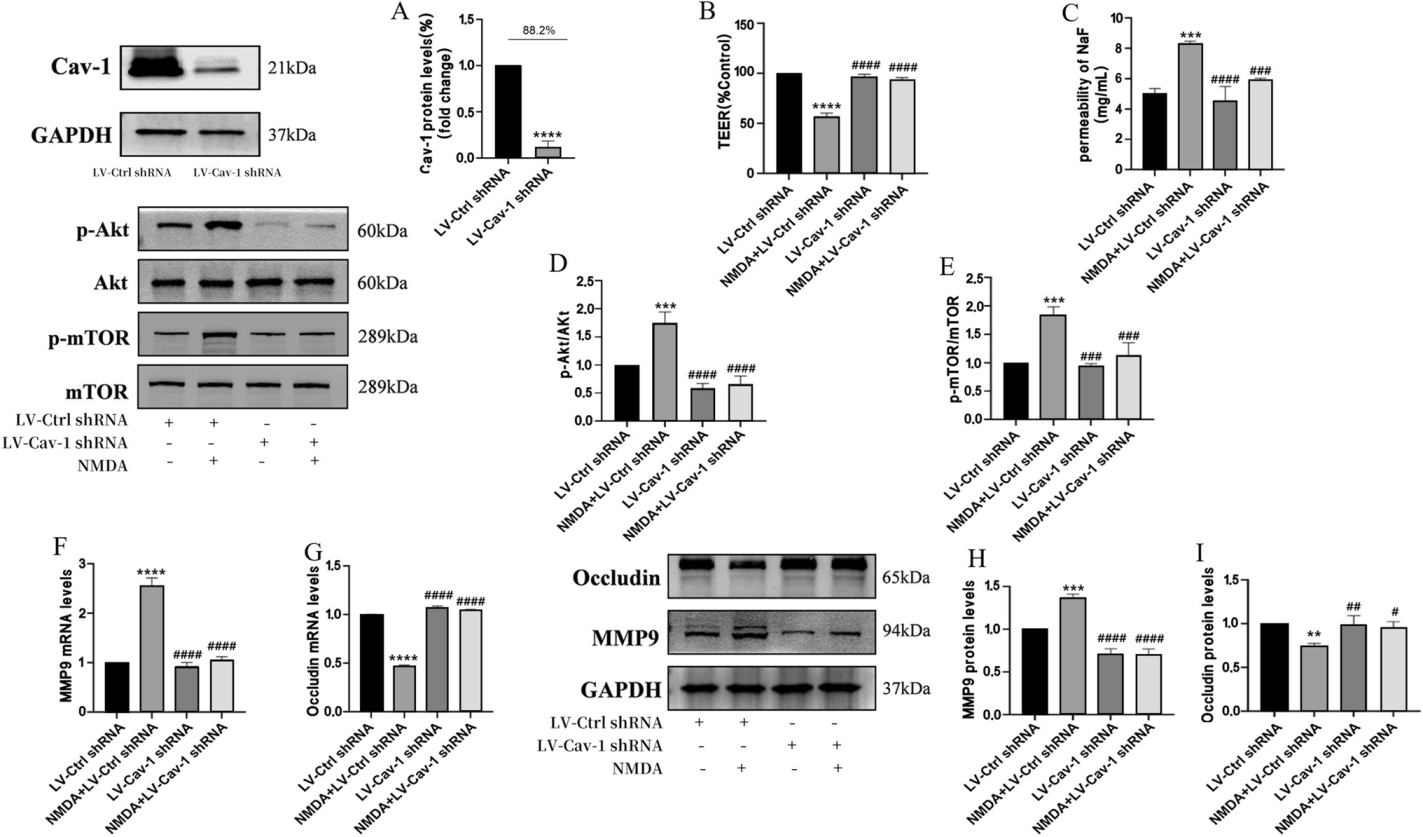
The Cav-1 inhibition rate of the stablemonoclonal cell lines was 88.2%, as detected by Western blot. Knock out of Cav-1 reversed NMDA-induced dysfunction in TEER and SF flux of BBB model in vitro and expressions of MMP9 and Occludin by the Akt/mTOR signal pathway*.* Silencing Cav-1 could restore decreased TEER after cells were exposed to NMDA (**B**). NMDA-induced increase SF flux was reversed by silencing Cav-1 (**C**). Protein expressions of p-Akt/Akt (**D**), p-mTOR/mTOR (**E**) were detected by Western blot assay. The mRNA and protein levels of MMP9 (F and H) and Occludin (G and I) were measured by RT-PCR and by Western blot, respectively. Each value represents the mean ± SD (*n* = 4). **p < 0.01, ***p < 0.001, ****p < 0.0001 versus LV-Ctrl shRNA group; #*p* < 0.05, ##*p* < 0.01, ###*p* < 0.001, ####*p* < 0.001, versus NMDA + LV-Cav-1 shRNA group

To further confirm that the effect of Cav-1 on NMDA-induced BBB dysfunction might be due to Akt/mTOR pathway activation, Cav-1 was silenced by short hairpin RNA in HBEC-5i (LV-Cav-1 shRNA). Lentivirus vectors without the Cav-1 sequence were used as RNA inference control (LV-Ctrl-shRNA group). The Cav-1 inhibition rate of the stable monoclonal cell lines was 88.2%, as detected by Western blot (Fig. 5A). Cav-1 depletion reversed the decrease of TEER and increase of SF flux after NMDA exposure (Fig. 5B, C). This led to a reduction in p-Akt and p-mTOR expression in the LV-Cav-1 shRNA group, compared to the LV-Ctrl-shRNA group (Fig. 5D, E). NMDA-induced expressions of MMP9 upregulation and Occludin degradation were diminished (Fig. 5F, G, H and I).

### Effects of Akt in NMDA-Induced changes of MMP9 and Occludin Blockade of Akt Activity Prevents BBB Disruption

**Fig. 6:**
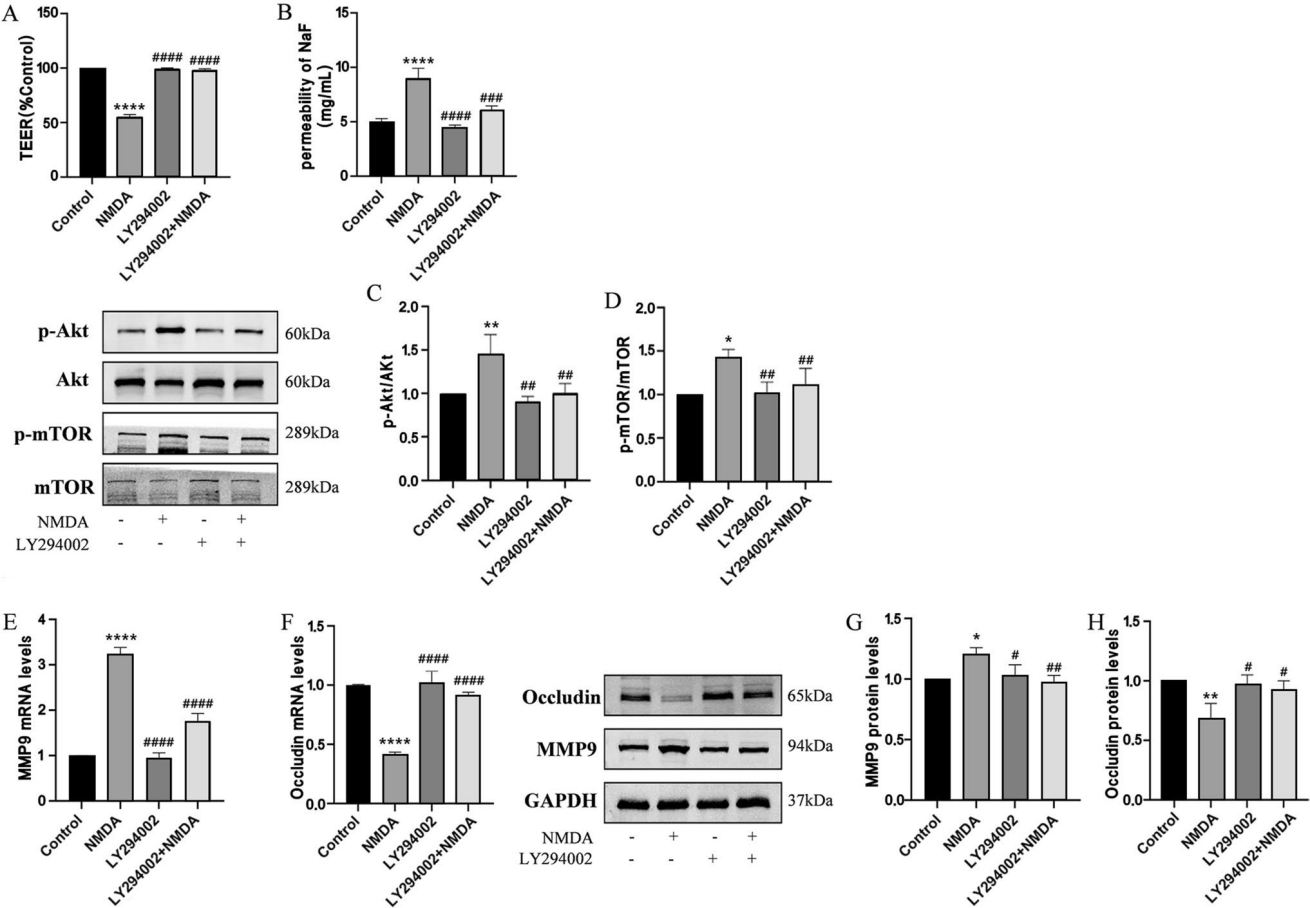
Inhibition of Akt reversed NMDA-induced dysfunction in TEER and SF flux of the BBB model in vitro and expressions of MMP9 and Occludin by the Akt/mTOR signal pathway*.* Pretreatment with Akt inhibitor LY294002 (10 μM) for 2 h could restore decreased TEER after exposed to NMDA (**A**). NMDA-induced increase SF flux was reversed by LY294002 (**B**). Protein expressions of p-Akt/Akt (**C**) and p-mTOR/mTOR (**D**) were detected by Western blot assay. The mRNA and protein levels of MMP9 (E and G) and Occludin (F and H) were detected by RT-PCR and by Western blot, respectively. Each value represents the mean ± SD (*n* = 4). *p < 0.05, **p < 0.01, ****p < 0.0001 versus control group; ##*p* < 0.01, ###*p* < 0.001, ####*p* < 0.001 versus NMDA group

The Akt/mTOR pathway undergoes a dramatic increase in signaling activity after injury, and has become a focus for drug development for the treatment of central nervous system injuries [34]. In order to address the role of Akt/mTOR in NMDA-stimulating BBB dysfunction, HBEC-5i were pretreated with Akt inhibitors (LY294002, 10 μM) for 2 h and then co-exposed to NMDA for 24 h. Pretreatment of cells with LY294002 could protect BBB function (Fig. 6A and B), decrease NMDA-induced augmentation in levels of phosphorylated Akt and mTOR (Fig. 6C and D), and diminish the increase of MMP9 and downregulation of Occludin (Fig. 6E, F, G and H).

## Discussion

Glutamate is the most abundant excitatory neurotransmitter in the CNS. Under pathological conditions, flooding of glutamate into the extracellular space can further open the BBB through NMDAR mediated Ca^2+^ overload on human microvascular endothelial cells and promote oxidative stress. The subsequent “vasogenic edema” plays a key role in cerebral edema of ischemic stroke [3]. In addition, NMDAR activity is associated with other neurological diseases, such as epilepsy, anti-NMDAR encephalitis, and Alzheimer's [35], suggesting the therapeutic potential of NMDAR modulators in these conditions. Our previous research, as well as other data, indicated that overstimulation of NMDAR on multiple cell lines of brain microvascular endothelial cells (ECV304, MCECs, BMEC, HBMEC) inhibited the expression of TJs such as Occludin, Claudin-5, and ZO-1 [4, 36]. The overstimulation also increased tyrosine phosphorylation and decreased threonine phosphorylation of Occludin [27]. In addition, NMDAR activation increased the expression of MMP9, which could degrade Occludin into inactive fragments [23] and increase BBB permeability via phosphorylation of myosin light chain [37]. To clarify the subcellular localization of GluN1 in HBEC-5i line, GluN1 was observed by laser confocal after immunofluorescence staining. These results shown that GluN1 was mainly distributed in the cytoplasm and membrane of HBEC-5i (Fig. 2). However, it is unclear if NMDAR activation affects the function of the BBB. In our study, decreased TEER and increased SF permeability in HBEC-5i-constructed BBB were found after NMDA exposure (Fig. 3A and B). The destruction of the BBB was also accompanied by the increase of MMP9 expression and Occludin degradation. Pretreatment with NMDAR noncompetitive inhibition (MK801) could reverse the changes of MMP9 and Occludin (Fig. 3F, G, H and I). This protective effect of MK801 on BBB function was also supported by other previous experimental results in vivo*.* MK801 pretreatment attenuated brain edema formation after middle cerebral artery occlusion (MCAO) in temporary focal cerebral ischemia [38] and restored BBB permeability after experimental diffuse brain injury [39]. Although MK801 has potential neuroprotective effects, it can block almost all the NMDAR in the CNS, resulting in toxicity and side effects such as schizophrenia-like symptoms, along with learning and memory impairment. At present, MK801 is widely applied to induce memory and learning impairment in preclinical studies [40].

Interestingly, elevations in the phosphorylation levels of Cav-1, Akt, and mTOR after NMDAR activation were observed in the current study. It is speculated that the Cav-1 related Akt/mTOR pathway may be responsible for the destruction of TJs. As the main component of caveolae, Cav-1 is generally expressed at the highest level in most cells, such as adipocytes, endothelial cells, fibroblasts, smooth muscle cells, and various epithelial cells [41]. Our study showed that Cav-1 was abundant in the membrane of HBEC-5i. There is no unified conclusion about the role of Cav-1 in different types of CNS diseases and cellular events. As a platform for signal transduction, the inhibition of Cav-1 and destruction of caveolae can reduce or eliminate the dysfunction of the BBB, or even have the opposite effect [42]. To further clarify the role of Cav-1 in NMDA-induced BBB dysfunction, Cav-1 inhibitor Daidzein was used to inhibit Cav-1. Inhibition of Cav-1 phosphorylation by Daidzein attenuated BBB destruction, restored Occludin expression, and reduced MMP9 (Fig. 4F, G, H and I). Furthermore, Daidzein did not change the total level of Cav-1. Although only about 5% of Cav-1 total protein can be phosphorylated by c-Src at tyrosine 14 site (Tyr-14) [43], the formation and endocytosis of caveolae depend on the conformational changes produced by Cav-1 phosphorylation [44]. It is believed that Cav-1 phosphorylation increases the density of caveolae on the surface of endothelial cells, promotes transcytosis of proteins, and leads to BBB disintegration [45]. Our previous data [46] found that in HIV-induced BBB injury, the regulation of Cav-1 affected the transcriptional induction of MMP-9 expression by affecting the Akt signaling pathway. Zou [47] et al. pretreated MCAO rats with electroacupuncture (EA) and antagonized the degradation of claudin-5 and Occludin, along with the expression of p-Cav1 and p-Akt. However, there was no significant difference between Cav-1 and Akt total protein. Those results demonstrated a close relationship between Cav-1 and Akt signaling. To further explore the role of Cav-1 in NMDA-stimulated BBB dysfunction and its potential mechanism, we used lentivirus as vectors to silence Cav-1 expression. Like the finding of inhibiting Cav-1 with Daidzein, knocking down Cav-1 significantly ameliorated NMDA-induced decreased TEER and increased SF permeability (Fig. 5B and C). Moreover, the alleviation of TJs destruction was linked to Akt/mTOR dephosphorylation (Fig. 6E, F, G and H). This data indicates that Cav-1 phosphorylation is involved in NMDAR activation mediated BBB breakdown, thus silencing Cav-1 could have a protective effect.

As a downstream effector of PI3K, Akt is a key regulator of cell survival signals. The interaction between hyperphosphorylated Akt and mTOR complex directly activates the Akt/mTOR signaling pathway [48]. This interaction widely participates in the physiological and pathological regulation of CNS, such as axon guidance, dendritic development, dendritic spine morphogenesis, and synaptic plasticity [49]. In our study, after treatment with NMDA, phosphorylation of Akt/mTOR in HBEC-5i was enhanced. MK801, Daidzein, and LY294002 were used to inhibit NMDAR, Cav-1, and Akt respectively. Then, the expression levels of p-Akt and p-mTOR were analyzed by WB assay (Figs. 3D, E, 4D, E, 5D, E and 6C and D). Our data showed that the alterations of p-Akt and p-mTOR were correlated with the expressions of MMP9 and Occludin. These outcomes demonstrated that Akt/mTOR might be a key downstream kinase stimulated by NMDA.

However, this study has several limitations. Although the application of BBB model in vitro can deeply study various physiological processes at the cellular and molecular levels, it needs to be validated again through in vivo experiments in complex structural components and microenvironments; BBB disruption is the result of complex interactions between multiple cells and multiple signaling pathways, and there may be crosstalk among other pathways in addition to the Cav-1/Akt/mTOR pathway.

## Conclusions

In summary, our study showed that NMDA-triggered damage in the integrity of the BBB could be restored by inhibition of NMDAR, Cav-1, Akt, and Cav-1 deletion. This protective effect may be associated with the regulation of the expression of MMP9 and Occludin via inhibiting the Cav-1 related Akt/mTOR pathway.

### Supplementary Information

Below is the link to the electronic supplementary material.

Supplementary file1 (PNG 82 KB)

## Data Availability

All data generated or analysed during this study are included in this published article.

## Abbreviations

NMDA: N-methyl-D-aspartate
Cav-1: Caveolin-1
BBB: Blood brain barrier
TEER: Transendothelial electrical resistance
SF: Fluorescein sodium
CNS: Central nervous system
TJs: Tight junctions
MMP9: Matrix metalloproteinase9
ROS: Reactive oxygen species
HBMECs: Human brain microvascular endothelial cells
PCR: Polymerase chain reaction
Pe: Permeability coefficient
qRT-PCR: Quantitative real-time polymerase chain reaction
MCAO: Middle cerebral artery occlusion
