# Supplementary figures and images for: Inhibiting Caveolin-1-Related Akt/mTOR Signaling Pathway Protects Against N-methyl-D-Aspartate Receptor Activation-Mediated Dysfunction of Blood–Brain Barrier in vitro
Source: Mol Neurobiol. 2023 Dec 8;61(7):4166–77. doi: 10.1007/s12035-023-03833-7 (PMC11236913; doi:10.1007/s12035-023-03833-7)

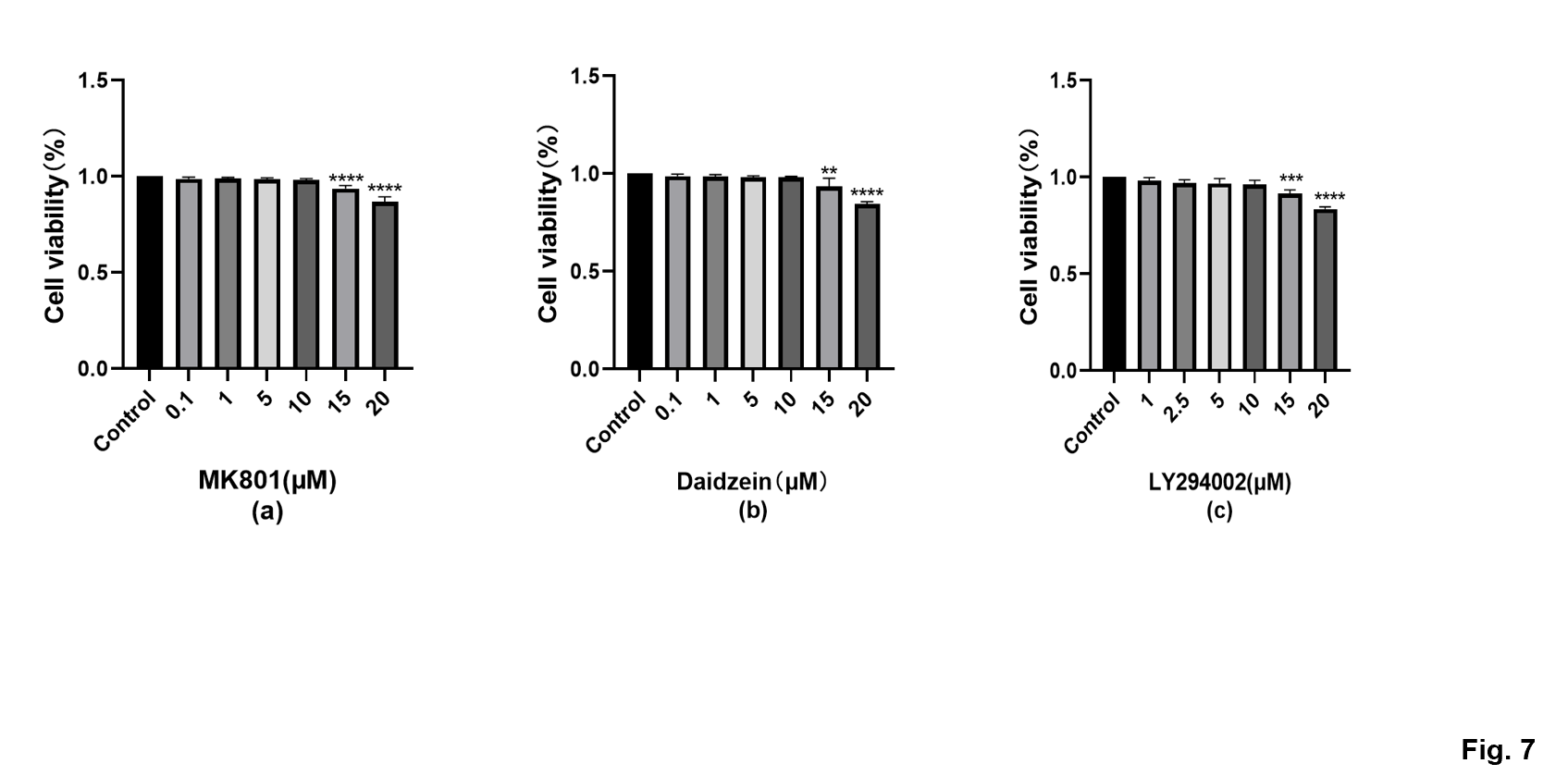

Supplement: Supplementary file 1 — Supplementary file1 (PNG 82 KB) [file 12035_2023_3833_MOESM1_ESM.png]
